# Paleoclimatic information recorded in fluid inclusions in halites from Lop Nur, Western China

**DOI:** 10.1038/s41598-017-16619-4

**Published:** 2017-11-27

**Authors:** Xiao-hong Sun, Yan-jun Zhao, Cheng-lin Liu, Peng-cheng Jiao, Hua Zhang, Chi-hua Wu

**Affiliations:** Ministry of Land and Resources (MLR) Key Laboratory of Metallogeny and Mineral Assessment, Institute of Mineral Resources, Chinese Academy of Geological Sciences (CAGS), Beijing, 100037 China

## Abstract

The homogenization temperature (T*h*) of primary fluid inclusions in halite can be used for paleoclimate interpretations. Lop Nur, in Central Asia, is an extremely arid zone where large amounts of glauberite were deposited from the late Middle to Late Pleistocene. This deposition was accompanied by formation of large-scale potash-bearing brines. However, quantitative paleotemperature data are still lacking, hindering reconstruction of Quaternary climate conditions and their control over potash formation. We measured the T*h* of inclusions in halite from the salt field and the top of Upper Pleistocene strata in Lop Nur. The maximum homogenization temperature (T*h*_MAX_) of inclusions in halite from the salt field was 41.1 °C, consistent with the maximum ambient temperature (43.4 °C) in the same period. The T*h*_MAX_ of inclusions in halite from the Upper Pleistocene strata ranged from 35.6 °C to 43 °C, where maximum air temperatures may have reached 37.9 °C to 45.3 °C. The results show that a hot and arid climate prevailed in Lop Nur at the end of the Late Pleistocene. Furthermore, changes of the brine chemical composition due to supply variations instead of climate change, may have caused glauberite deposition to cease at the end of the Late Pleistocene.

## Introduction

The environmental evolution of lake sedimentary records is an important field in the study of past global changes (PAGES)^1^. Saline lakes, as the final product of lake evolution, are a key aspect of research into paleoclimates, paleoenvironments, and future global changes^2–6^. The Lop Nur saline lake, located in northwestern China, is one of the largest playa lakes in the world. It was once China’s largest inland lake, around which the renowned civilization of the ancient Loulan Kingdom emerged. It has been studied by scientists and explorers around the world since the end of the nineteenth century^7,8^. In addition, the Lop Nur saline lake is the second largest potash mineralization area in China^9^, and has been the largest source of high-quality potassium sulfate fertilizer in the world. Potash deposits are formed as a result of coupling among particular provenances, tectonics, and climatic conditions^10^. Among these factors, high temperature may have served as a catalyst for the precipitation of potassium from the saline lake water^11^.

The climate is extremely arid in the Lop Nur area. The region has an average annual rainfall of approximately 20 mm and average annual evaporation of approximately 3,000 mm^7^. Over the past 20 years, many studies have investigated sedimentary characteristics^12–14^, salt lake evolutionary history^9,15^, the formation mechanism of potassium-rich brine^10,16–19^, and paleoclimate and environmental evolution^20–31^ in Lop Nur. However, minimal research has been conducted on quantitative reconstructions of paleotemperature and its effect on potassium formation in Lop Nur. Understanding paleoclimate change in Lop Nur, a microcosm of arid environments, is vital for a range of topics, including the environmental evolution of Central Asia, the demise of the ancient Loulan kingdom, and the genesis of potassium-rich brine.

Quantitative paleoclimatic information has been indirectly obtained from biological and geochemical methods, including palynological assemblages^32,33^, organic biomarkers^34–37^, chironomids^38–41^, and hydrogen isotopes^42–44^. However, it is difficult to obtain accurate paleotemperature data from evaporite sediments in a salt lake using these methods. Theoretically, fluid inclusions are excellent geothermometers. Homogenization temperatures (T*h*) obtained from primary single-phase (liquid) fluid inclusions in halite through the so-called cooling nucleation method can reflect paleobrine temperatures during salt-forming periods^45–47^. Additionally, the paleobrine temperature is generally close to the environmental paleotemperature in shallow water^48^.

The cooling nucleation method has been widely used for paleotemperature reconstruction in geological periods, including the Precambrian^49^, Silurian^50,51^, Permian^52,53^, Cretaceous^54,55^, Paleogene^56,57^, and Quaternary^3,46^. In this study, following detailed petrographic observations of halite samples from the salt field and a drill core in Lop Nur, the cooling nucleation method was used to obtain the homogenization temperature of fluid inclusions. We then considered the implications of these homogenization temperatures for paleoclimatic conditions in Lop Nur at the end of the Late Pleistocene.

## Results

### Site descriptions and material

**Figure 1:**
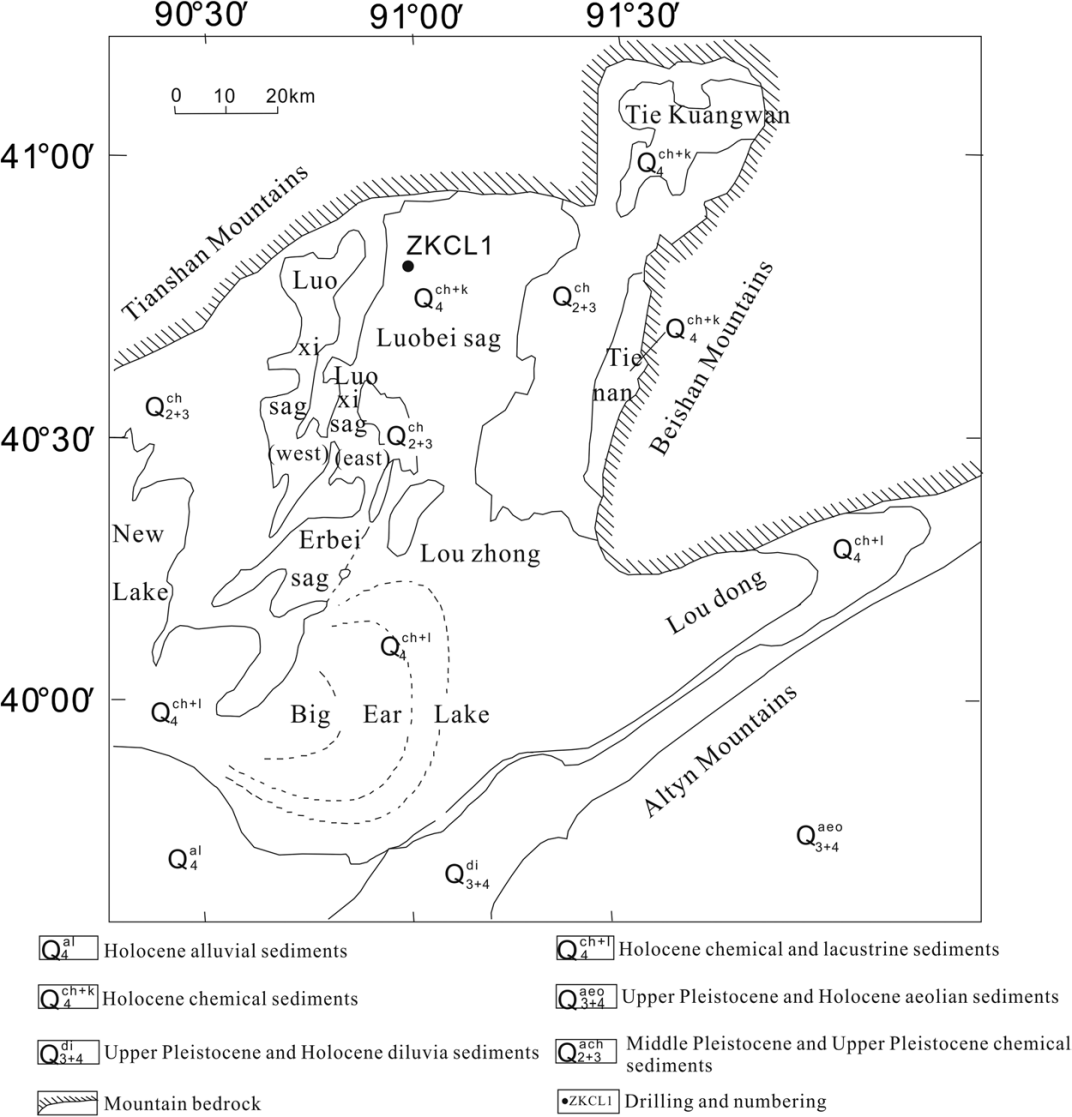
Schematic geological map of Lop Nur in Xinjiang, China, and the location of core ZKCL1 (modified after Liu *et al*.^17^).

Lop Nur (39°40′ – 41°20′N, 90°00′ – 91°30′E), covering an area of approximately 20,000 km^2^, lies in the eastern part of the Tarim Basin. It is bordered to the north by the Tianshan Mountains, to the west by the Taklamakan Desert, and to the southeast by the Altun Mountains (Fig. 1). The Quaternary Himalayan movement caused the western Tarim Basin to uplift and the eastern part to subside. As a result, Lop Nur is situated at the convergence of sediments and salts throughout the Tarim Basin^58^, and is the lowest part of the basin (780 to 795 m above sea level). Tectonically, Lop Nur is a sub-basin of the Tarim Basin. It is situated at the junction of the Tianshan Orogenic Belt and Kunlun Orogenic Belt^9,10^. Faults inside and around the Lop Nur depression primarily include the Kuruk Tag Fault, Altyn Tagh Fault, Kongqi River Fracture, Saisike Fracture, Shule River Fault, and southern and eastern Lop Nur Faults.

Lop Nur was a unified lake from the Early Pleistocene to the earliest stage of the Middle Pleistocene^59^. The lake environment changed into brackish water, in which thin-layered and dispersed gypsum were deposited. Strong uplift occurred in the northern part of Lop Nur from the end of the Middle Pleistocene to the Later Pleistocene, resulting in formation of a series of sub-depressions, such as Luobei Sag, partitioning the previously integrated Lop Nur. Luobei Sag is one of the largest sub-depressions. It is characterized by large amounts of glauberite with relatively decreased gypsum. The brackish lake environment then changed into a saline lake environment. Thinly layered halite, polyhalite, and bloedite were deposited at the end of the Later Pleistocene. By the Holocene, the Luobei Sag and other sub-depressions had completely evolved into saline lake environments. Thinly layered halite and a small amount of other salt minerals, including polyhalite, carnallite, sylvite, epsomite, and kainite, were deposited^9^.

**Figure 2:**
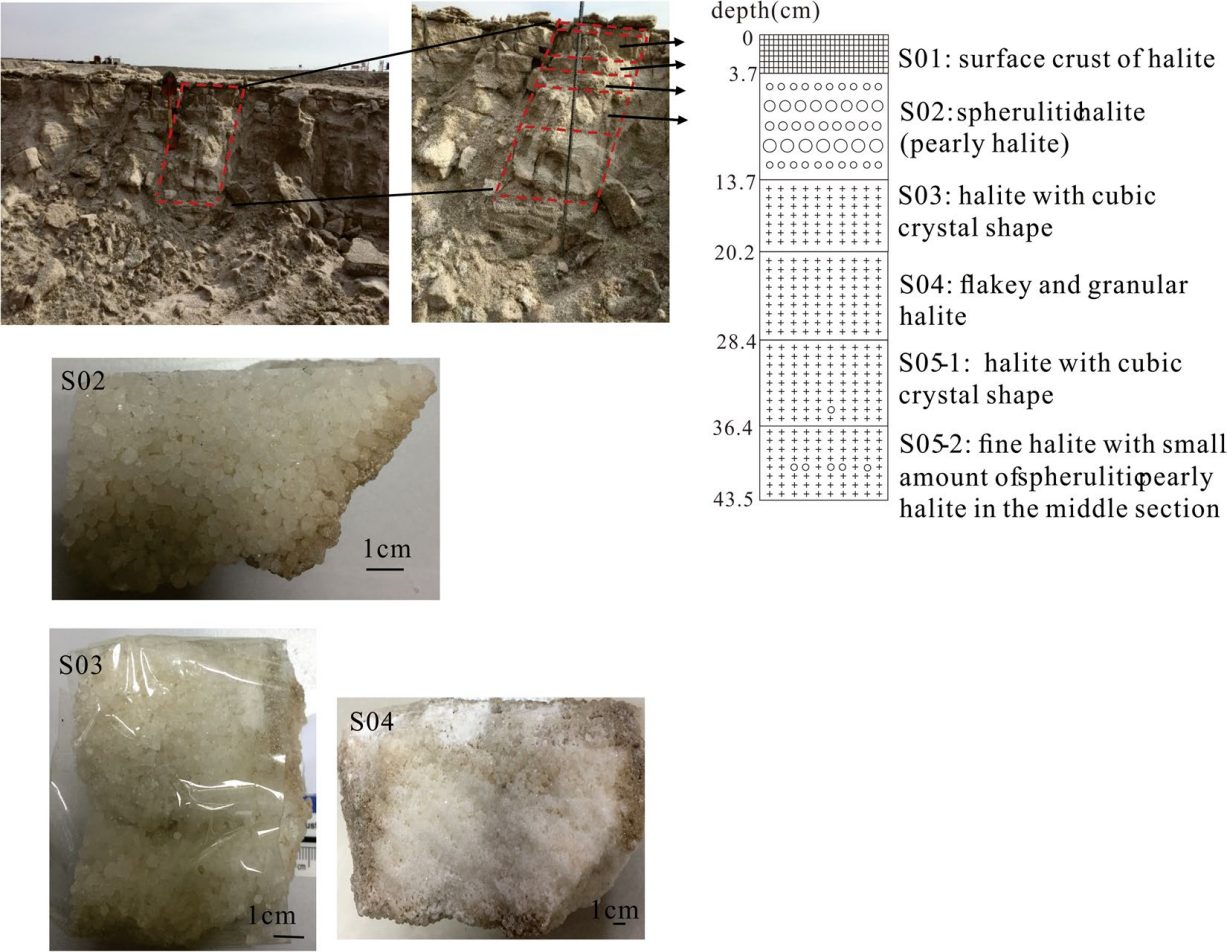
Schematic representation of the 43.5-cm deep pit, and photomicrographs of the samples.

The samples used in this study were modern halite from the salt field in the center of the Lop Nur depression, and Latest Pleistocene halite from a 104.12-m core from Luobei Sag. The salt field was abandoned in 2009. Five halite samples, deposited in 2008 based on observations, were collected from a 43.5-cm deep pit (Fig. 2). From top to bottom, the layers were: a surface halite crust (S01) from 0 to 3.7 cm; white spherulitic halite (pearly halite) (S02) from 3.7 to 13.7 cm, with particle sizes of 1 to 4 mm; white halite (S03) from 13.7 to 20.2 cm, with particle sizes of 1 to 4 mm; white halite with flaky and granular shapes (S04) from 20.2 to 28.4 cm, and particle sizes of 1 to 4 mm; and white halite (S05) from 28.4 to 43.5 cm, with particle sizes of 1 to 3 mm.

A homogenization temperature analysis was performed on all samples, except S01. Moreover, S05 was divided into two samples during the analysis: S05-1 and S05-2. S05-1 was from 28.4 to 36.4 cm, with good halite crystal shapes and minimal pearly halite. S05-2 was from 36.4 to 43.5 cm, and mainly consisted of fine halite with a small amount of spherulitic pearly halite in the middle section.

**Figure 3:**
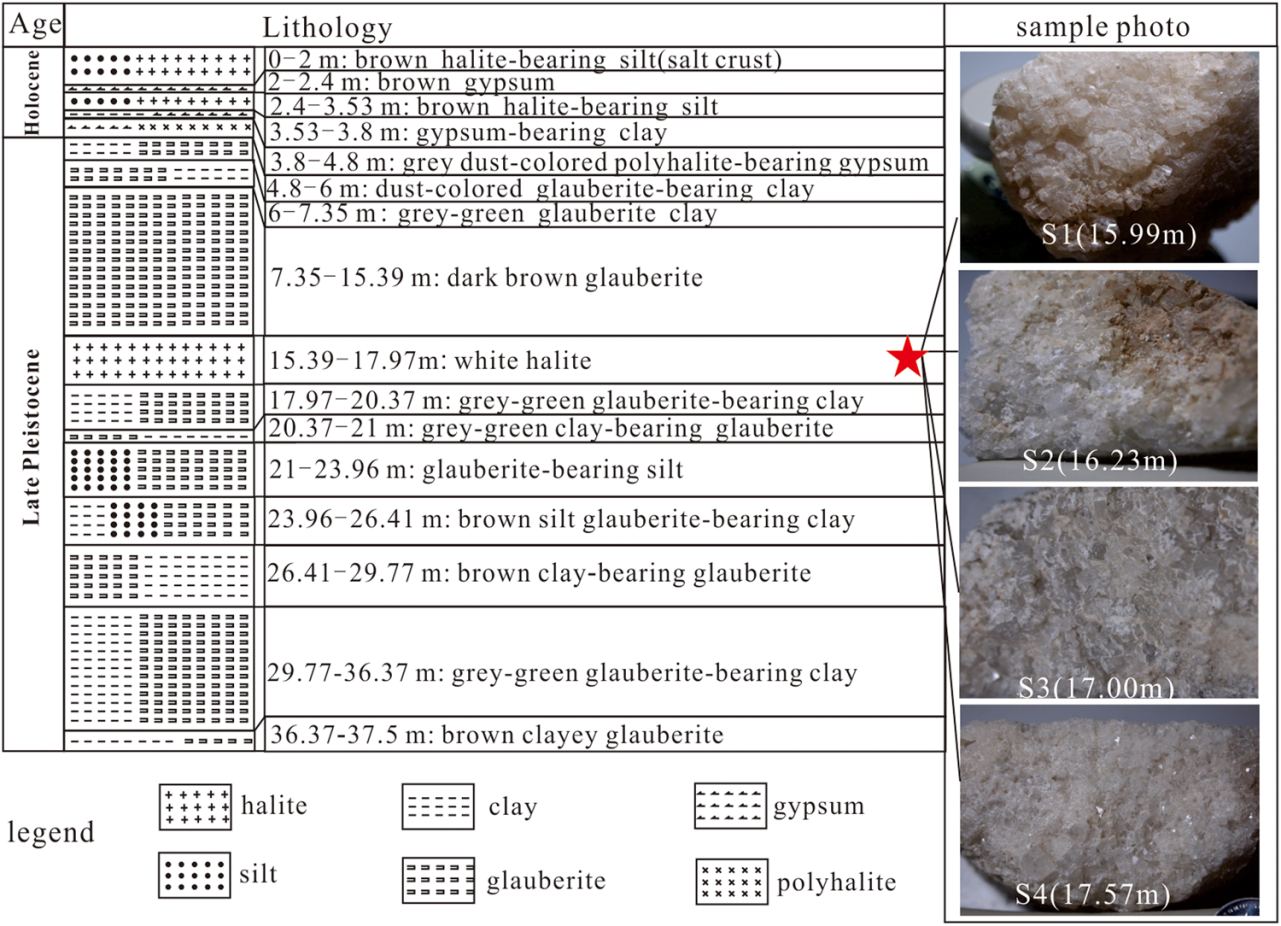
Core histograms of core ZKCL1 from 0 to 37.5 m, and images of the core samples.

Many cores were drilled in the Lop Nur mining region for exploration and development of commercial potash deposits. The core investigated in this study (ZKCL1) was drilled in the northwest of the Luobei depression at 40°48′17.236″N, 90°59′00.225″ E to a depth of 104.12 m. Based on the comparisons with other published cores in lithology^9^, the depth of the boundary between the Late Pleistocene and Holocene was approximately 4.8 m, and characterized by a basal needle gypsum layer. The lithology and cyclic sequence of sediments in the core are shown in Fig. 3.

In total, four halite samples were collected from the core for homogenization temperature analysis at 15.99 m (s1), 16.23 m (s2), 17.0 m (s3), and 17.57 m (s4). All samples were obtained from the top of the Upper Pleistocene strata (Fig. 3). Visual analysis indicated that they were comprised of white halite, which has the crystal form of idiomorphic cubes with fine to medium grains, a massive structure, and 3% intergranular porosity.

**Figure 4:**
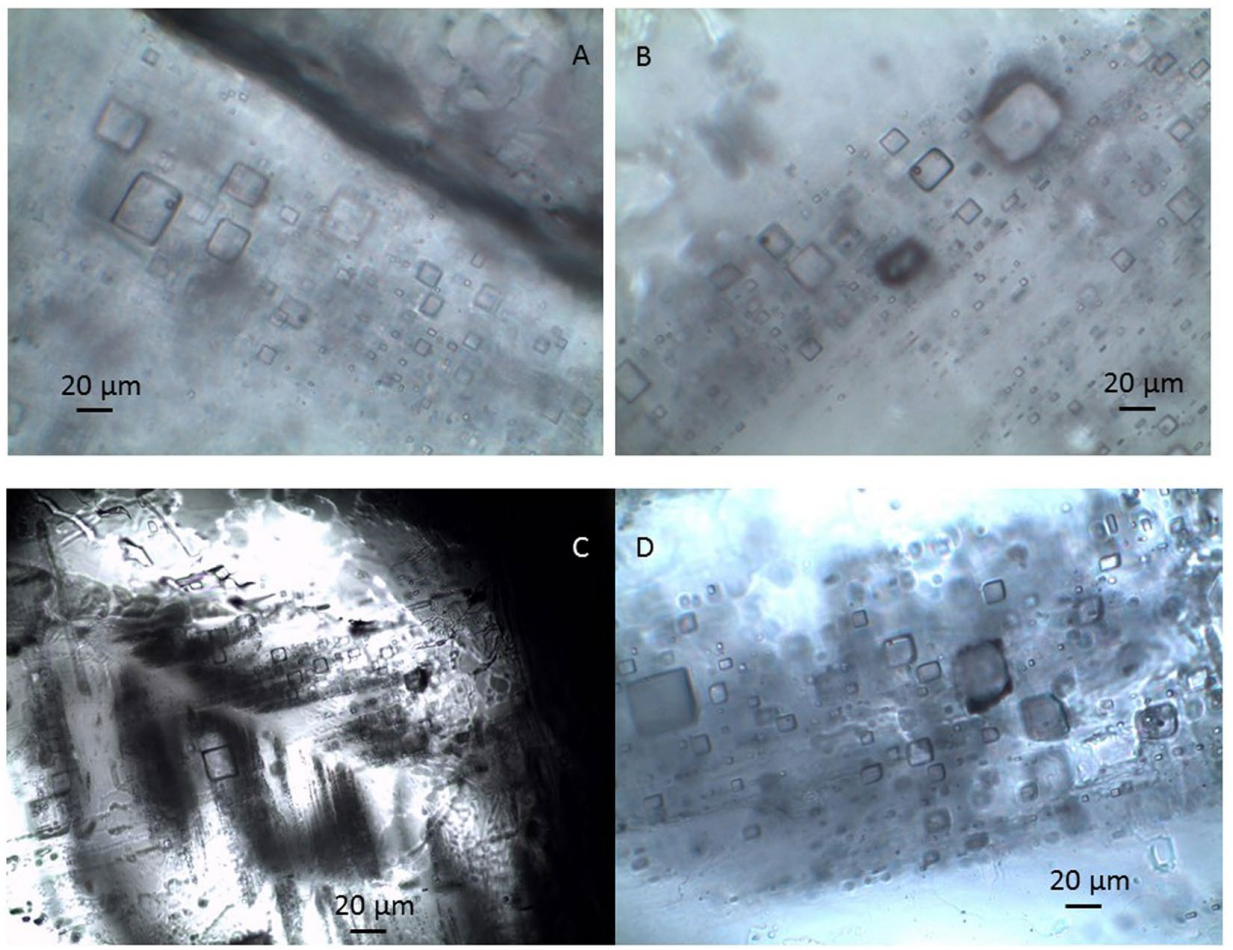
Primary fluid inclusions in salt-field halite and drill-core halite in Lop Nur. A photomicrograph of: (**A**) and (**B**) primary fluid inclusions banding in cumulate halite of samples from the salt field, (**C**) primary fluid inclusions in chevron halite of a sample from the drill core, and (**D**) primary fluid inclusions banding in cumulate halite of a sample from the drill core.

Primary halite is characterized by cumulate crystals, usually formed at the air–water interface, and chevron crystals formed at the bottom of saline lakes^60^. These crystals commonly contained well-defined fluid inclusion banding parallel to the halite crystal growth faces. Primary fluid inclusions in the salt-field halite were trapped within cumulate crystals, while primary inclusions in the drill core were trapped within chevron or cumulate crystals (Fig. 4). The observed halite inclusions were predominantly square. Generally, the primary fluid inclusions were dominated by single-phase liquid inclusions, and the size ranged from 3 to 80 μm in diameter. Two- and three-phase inclusions were rarely observed under laboratory temperatures.

### T*h* record

**Figure 5:**
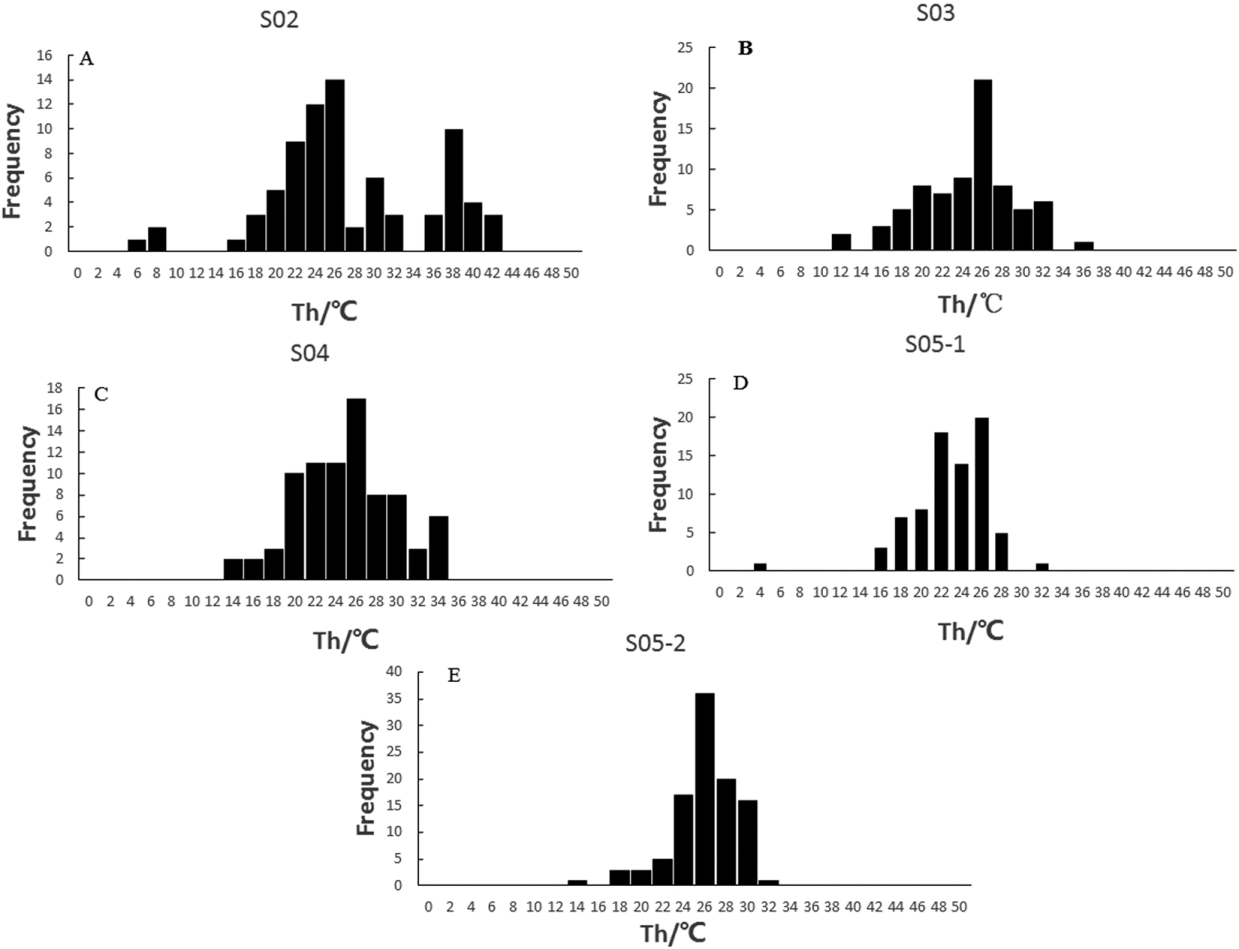
Histogram of homogenization temperatures plotted against the number of halite fluid inclusions in the salt field. (**A**) Sample S02, 40 T*h* data from primary fluid inclusions; (**B**) sample S03, 75 T*h* data from primary fluid inclusions; (**C**) sample S04, 81 T*h* data from primary fluid inclusions; (**D**) sample S05-1, 102 T*h* data from primary fluid inclusions; and (**E**) sample S05-2, 77 T*h* data from primary fluid inclusions.

**Figure 6:**
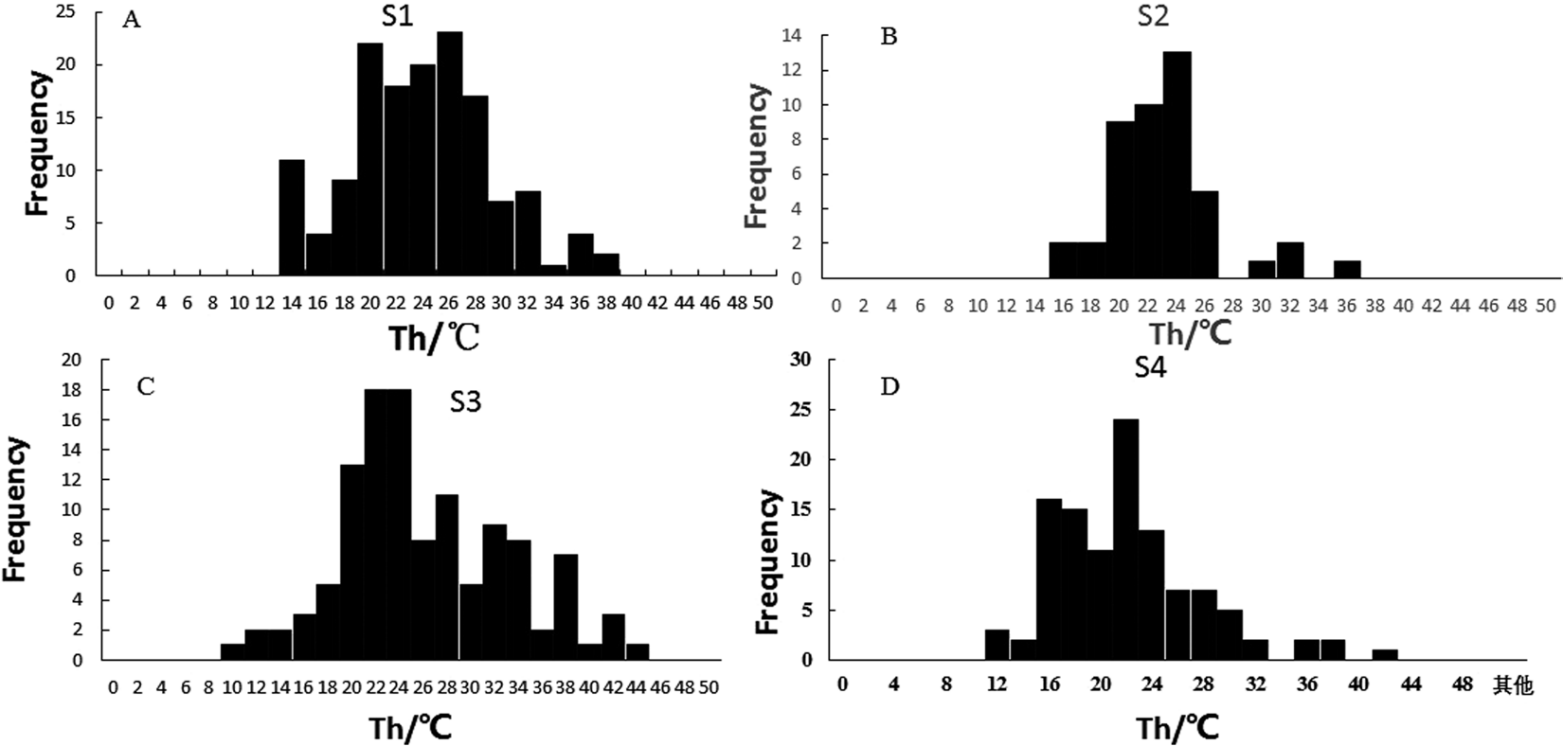
Histogram of homogenization temperatures plotted against the number of halite fluid inclusions in Upper Pleistocene strata in the Luobei depression. (**A**) Sample S1, 146 T*h* data from primary fluid inclusions; (**B**) sample S2, 45 T*h* data from primary fluid inclusions; (**C**) sample S3, 117 T*h* data from primary fluid inclusions; and (**D**) sample S4, 110 T*h* data from primary fluid inclusions.

We measured homogenization temperatures (T*h*) in 413 primary fluid inclusions in five halite samples from the abandoned salt field. The recorded T*h* data are summarized in Table 1 and Fig. 5. In total, 418 T*h* data were obtained from four halite samples in the Upper Pleistocene strata of the Luobei depression, with a maximum T*h* of 43 °C and a minimum T*h* of 9.5 °C (Table 2 and Fig. 6).

**Table 1:** Summary of homogenization temperatures of halite fluid inclusions in the salt field.

Sample	Depth (cm)	number	T*h*_MAX_ (°C)	T*h*_MIN_ (°C)	T*h*_AVG_ (°C)	T*h*_RANGE_ (°C)
S02	3.7–13.7	78	41.1	4.6	26.51	36.5
S03	13.7–20.2	75	35.7	11.7	23.58	24
S04	20.2–28.4	81	32.5	13.5	24.16	19
S05-1	28.4–35.5	102	30.1	12.9	24.98	17.2
S05-2	35.5–43.5	77	31.6	2.9	22.07	28.7

**Table 2:** Summary of homogenization temperatures of halite fluid inclusions in Upper Pleistocene strata in the Luobei depression.

Sample	Depth (m)	number	T*h*_MAX_ (°C)	T*h*_MIN_ (°C)	T*h*_AVG_ (°C)	T*h*_RANGE_ (°C)
S1	15.99	146	36.9	12.7	23.03	24.2
S2	16.23	45	35.6	15.7	22.16	19.9
S3	17	117	43	9.5	25.21	33.5
S4	17.57	110	40.2	10.2	21.45	30

## Discussion

The tectonic activity in the study area has been relative weak since the late Pleistocene^9^. There is no obvious deformation characteristics based on our macro- and microscopic observations of the halite samples. Fluid inclusions of variable sizes were selected for micro-thermometry to evaluate whether the large inclusions were subjected to re-equilibration. Our results do not show a close relationship between inclusion size and homogenization temperatures (Fig. S1). It suggests that fluid inclusion samples were not altered by thermal re-equilibration or other geological processes. Therefore, the homogenization temperatures in this study are reliable and representative of the temperature of salt precipitation.

Halite is a major evaporate mineral on the Earth’s surface. Two types of primary fluid inclusion bands typically develop along the cleavage plane during the halite crystal formation process^45,46,52,61^. In cumulate crystals, they form at the air–water interface; in chevron crystals, they form at the bottom of saline lakes. Chevron-type halite is typically deposited in shallow water environments^52^. Primary fluid inclusions in both cumulate and chevron halite have a similar T*h*_MAX_, which can be used to interpret paleoenvironmental conditions in shallow water^48^. It has been suggested that T*h*_MAX_ values of fluid inclusions in modern halite samples from Death Valley, CA (34 °C) are consistent with maximum brine temperatures during halite precipitation (34.4 °C), and correlate well with average maximum air temperatures (31.3 °C)^46^.

Fluid inclusions in the salt-field halite occurred in cumulate crystals (Fig. 4), indicating that the T*h*_MAX_ of single liquid-phase inclusions can reflect both brine temperatures during halite precipitation and surface air temperatures. Examination of sample S02 indicated the presence of pearly halite (Fig. 2). Two important aspects of the genesis of pearly halite should be noted^62^. Firstly, halite crystallized out of the evaporating brine lake when sodium chloride reached supersaturation. Pearly halite may have formed by continual rolling and growth below the lake terrace under the action of wind and waves. Secondly, desalinated lake water may have eroded halite terraces under the action of wind and waves. The halite crystals that were washed away may have been slightly eroded by the lake water, and the pearly halite may have formed through continual rolling. In short, pearly halite likely formed by the rolling process. It was perennially windy in the lake area, providing good hydrodynamic conditions for the formation of pearly halite.

Furthermore, there has been an ongoing drought in China’s Tarim Basin since the Pleistocene, especially during the Holocene. Lop Nur has the typical characteristics of a continental arid climate, including low precipitation, high evaporation, a large diurnal temperature difference, and strong wind power subject to constraints of the regional environment^9^. Northeast winds prevail in the study area. In 2008, the maximum wind speed reached 21.8 m/s in May, and the maximum temperature (43.4 °C) occurred in June (Table S1).

The pearly halite (sample S02) must have formed in May or June 2008 (slightly later than the date of the maximum wind speed) under the influence of strong winds. The recorded T*h* in primary fluid inclusions from sample S02 ranged from 4.6 °C to 41.1 °C, with a maximum T*h* of 41.1 °C, which was consistent with the average maximum air temperature in June (43.4 °C) in Lop Nur. There was no pearly halite in samples S03 or S04, and only a small amount in sample S05. The cumulative thickness from sample S05 to sample S02 was 39.8 cm, which was consistent with the observed annual deposition thickness (approximately 40 cm). The recorded T*h*_MAX_ of single-phase (liquid) fluid inclusions in the salt-field halite was 31.6 °C (S05), 32.5 °C (S04), 35.7 °C (S03), and 41.1 °C (S02), which reflected seasonal variations (autumn, winter, spring, and summer, respectively).

The T*h*_MAX_ of the single liquid phase inclusions represents the highest air temperature during halite deposition based on the relationship between surface air temperature and T*h* (a temperature difference of approximately 2.3 °C) obtained from halite fluid inclusions in the salt field at Lop Nur. Given the recorded T*h*_MAX_ of fluid inclusions in the Upper Pleistocene strata from the Luobei depression (35.6 °C to 43 °C, Table 2), we inferred that paleotemperatures during Upper Pleistocene halite deposition may have reached 37.9 °C to 45.3 °C. Some researchers have suggested a westerly pattern of climate change, with a cold–humid and warm–dry climate dominating the Late Pleistocene in the Xinjiang area^22,23,28,31,63–66^. Our results showed that a hot and arid climate prevailed in Lop Nur during the Late Pleistocene, which was consistent with the paleoclimatic conditions revealed by the T*h* of fluid inclusions in glauberite from other studied cores in the Luobei depression (including ZK1200B, ZK1608B, ZK0700, ZK1611, and ZK0300)^67^.

Based on the distribution of evaporite minerals in the Lop Nur strata^9^, gypsum deposition occurred during the Early–Middle Pleistocene, and glauberite deposition occurred from the end of the Middle Pleistocene to the Late Pleistocene in the northern part of Lop Nur. Thinly layered halite, polyhalite, and bloedite were deposited at the end of the Late Pleistocene. The Lop Nur salt lake was desalinated in the Holocene with needle gypsum as the marker bed. After needle gypsum deposition, halite was deposited. It is not clear why glauberite deposition ceased at the end of the Late Pleistocene. Glauberite is a typical warm-phase evaporite mineral. The higher the temperature, the more favorable the conditions for glauberite precipitation^67^.

Liu *et al*.^14^ concluded that the coupling of continuous drying and variation in the supply source may have caused the complex chemical sedimentary sequence in the Lop Nur salt lake. The values of T*h* obtained from halite fluid inclusions showed that a hot and arid climate prevailed at Luo Nur at the end of the Late Pleistocene, which was consistent with the paleoclimatic conditions of glauberite formation^67^, as well as those indicated by pollen, magnetic susceptibility, and other environmental proxies^22,23,31^. Therefore, changes in the chemical composition of the brine due to supply source variations instead of climate change, may have caused glauberite deposition to cease at the end of the late Pleistocene.

An arid climate is one of three necessary conditions for potash formation^10^. Liu *et al*.^28^ conducted high-resolution multi-proxy analyses using materials from a well-dated pit section (YKD0301) in the center of Lop Nur. They showed that Lop Nur experienced a progression through a brackish lake, saline lake, slightly brackish lake, saline lake, brackish lake, and playa due to climatic changes over the past 9,000 years. Potassium enrichment in Lop Nur may have occurred under these alternating conditions.

Four tectonic events have elevated the Tibetan Plateau since 2.8 Ma BP^68,69^. Since 30 ka BP, this uplift intensified, and greatly impacted the evolution of altiplano saline lakes^1^. The climate pattern in arid areas of northwestern China is very different from that in eastern monsoon areas. The climate in the Tarim Basin is cold and dry in the winter, and hot and arid in the summer. A previous study showed that abrupt cold/warm events in the northern hemisphere correspond to lake sediment records in Lop Nur, Xinjiang^23^, indicating that environmental evolution in arid areas of northwest China is also influenced by global climate change on millennial-centennial scales.

However, there are insufficient quantitative reconstructions of the paleoclimate because traditional proxies in the saline lake (e.g., palynological assemblages, organic biomarkers) are limited owing to the high salinity of the sediments. Using the homogenization temperature of fluid inclusions in the evaporate minerals is therefore a useful method for studying the effect of climate change on potash formation in Lop Nur.

## Methods

During sample preparation, it was important to avoid dissolution and overheating. Halite samples were separated into fragments with thicknesses of 0.5 to 1 mm using a hammer and chisel along cleavage planes. Detailed petrographic studies were then conducted on each halite fragment sample to document individual primary (liquid) fluid inclusions. The occurrence and morphology of each primary fluid inclusion were observed and photographed. All halite samples were placed in an airtight plastic box, and desiccant was added for moisture protection. In this study, we utilized the cooling nucleation method outlined in previous studies^46,48,49,52,54–57,70^. Samples were placed in a Haier freezer for one to two weeks at a stable temperature of −18 °C. The homogenization temperature was measured after the single-phase fluid inclusions were frozen to nucleate bubbles. Samples removed from the freezer were quickly placed in a Linkam THMSG600 heating/cooling stage and cooled rapidly to −18 °C. Then, the heating stage was warmed at a rate of 0.5 °C/min up to a temperature of 15 °C. Thereafter, the rate was lowered to 0.1 °C/min until all artificially nucleated vapor bubbles had disappeared (homogenized).

## Electronic supplementary material

Supplementary Information
