# Supplementary material for: Paleoclimatic information recorded in fluid inclusions in halites from Lop Nur, Western China
Source: Sci Rep. 2017 Nov 27;7:16411. doi: 10.1038/s41598-017-16619-4 (PMC5703717; doi:10.1038/s41598-017-16619-4)
Supplement: Supplementary file 1 — Supplementary Information [file 41598_2017_16619_MOESM1_ESM.doc]

Supplementary Information for

**Paleoclimatic information recorded in fluid inclusions in halites from Lop Nur, Western China**

**This file includes:**

**Table S1**

**Figure S1**

**Table S1**  Lop Nur meteorological conditions in 2008^*^

| Temperature（ºC） | | | | | Wind speed（m/s） | | |
| --- | --- | --- | --- | --- | --- | --- | --- |
| Mean temperature | Maximum temperature | Date of maximum temperature appeared | Minimum temperature | Date of minimum temperature appeared | Minimum wind speed | Maximum wind speed | Date of maximum wind speed appeared |
| 13.8 | 43.4 | June 3 | –22.5 | January 30 | 4.3 | 21.8 | May 2 |

^*^ Dependent on Luozhong meteorological station of SDIC Xinjiang Lop Nur Potash Co., Ltd.

**Figure S1 Histogram of homogenization temperatures plotted against size of inclusions**
